# Cobalt Ferrite Nanorods Synthesized with a Facile “Green” Method in a Magnetic Field

**DOI:** 10.3390/nano14060541

**Published:** 2024-03-20

**Authors:** Alexander L. Kwiatkowski, Petr V. Shvets, Ivan S. Timchenko, Darya E. Kessel, Elizaveta D. Shipkova, Konstantin I. Maslakov, Ivan A. Kuznetsov, Dmitry A. Muravlev, Olga E. Philippova, Andrey V. Shibaev

**Affiliations:** 1 Physics Department, Lomonosov Moscow State University, Leninskije Gory 1-2, 119991 Moscow, Russia; ivan.s.timchenko@gmail.com (I.S.T.); dkshmr@yandex.ru (D.E.K.); shipkova_liza@mail.ru (E.D.S.); pusido0907@yandex.ru (I.A.K.); mit89-angel@yandex.ru (D.A.M.); phil@polly.phys.msu.ru (O.E.P.); 2 REC “Functional Nanomaterials”, Immanuel Kant Baltic Federal University, Aleksandra Nevskogo St., 14, 236041 Kaliningrad, Russia; pshvets@kantiana.ru; 3 Chemistry Department, Lomonosov Moscow State University, Leninskije Gory 1-3, 119991 Moscow, Russia; nonvitas@gmail.com; 4 Chemistry Department, Karaganda E.A. Buketov University, University Street 28, Karaganda 100028, Kazakhstan

**Keywords:** anisotropy, cobalt ferrite, co-precipitation, crystal growth, magnetic field, nanorods

## Abstract

We report a new facile method for the synthesis of prolate cobalt ferrite nanoparticles without additional stabilizers, which involves a co-precipitation reaction of Fe^3+^ and Co^2+^ ions in a static magnetic field. The magnetic field is demonstrated to be a key factor for the 1D growth of cobalt ferrite nanocrystals in the synthesis. Transmission electron microscopy (TEM), X-ray diffraction (XRD), and Raman spectroscopy are applied to characterize the morphology and structure of the obtained nanoparticles. According to TEM, they represent nanorods with a mean length of 25 nm and a diameter of 3.4 nm that have a monocrystalline structure with characteristic plane spacing of 2.9 Å. XRD and Raman spectroscopy confirm the spinel CoFe_2_O_4_ structure of the nanorods. After aging, the synthesized nanorods exhibit maximum saturation magnetization and coercivity equal to 30 emu/g and 0.3 kOe, respectively. Thus, the suggested method is a simple and “green” way to prepare CoFe_2_O_4_ nanorods with high aspect ratios and pronounced magnetic properties, which are important for various practical applications, including biomedicine, energy storage, and the preparation of anisotropic magnetic nanocomposites.

## 1. Introduction

In recent years, high attention has been attracted to the synthesis and investigation of magnetic nanomaterials [1,2,3], which can be used in many areas, such as the preparation of magnetic nanocomposites and gels [4,5], soft robotics [6], energy storage devices [7], green energy production [8], and various biomedical applications [9,10], including hyperthermia [11], magnetic resonance imaging [12], the development of magnetically responsive industrial systems [13], and so forth. For such nanomaterials, anisotropic (cylindrical, plate-like, etc.) magnetic nanoparticles (NPs) are of high interest [14,15,16] because of their enhanced magnetic properties [17], anisotropy of magnetism, a larger area of the locally induced magnetic field in comparison to nanospheres, etc. [10], as well as their ability to impart anisotropy to nanocomposite materials.

Various methods for synthesis of elongated magnetic NPs have been described, including solvothermal [18], hydrothermal [19], sol–gel [20] or co-precipitation [21] reactions, ultrasound treatment [22], synthesis in the presence of polymers [23] or surfactants [24,25,26], modification of the crystal structure of pre-synthesized rod-like particles [12], etc. In most of these approaches, either a template or a stabilizer is used to provide the growth of NPs in one direction. After synthesis, the surface of NPs usually remains covered with the stabilizer, which may complicate its further modification with organic or inorganic compounds.

Recently, the use of a magnetic field as a template for the 1D growth of magnetic NPs was proposed [27,28,29,30,31,32,33]. For instance, a facile and “green” method for the synthesis of elongated magnetite (Fe_3_O_4_) nanoparticles was elaborated, which consists of the co-precipitation of Fe^3+^ and Fe^2+^ ions in a magnetic field [30,31]. This method allowed obtaining cylindrical Fe_3_O_4_ NPs of different lengths of up to 150 nm and magnetizations of ca. 29 emu/g. A growth mechanism was proposed, which includes the appearance of small spherical “seed” nanoparticles at the first reaction stage, which then self-assemble into a rod in the external magnetic field and fuse to form a cylindrical NP [32]. The magnetic field was further applied for the synthesis of several 1D nanomaterials, mostly nanowires [33].

Most of the approaches for anisotropic NP synthesis described above are rather well developed for iron oxides, such as magnetite or maghemite. It seems reasonable to apply this method to the preparation of other kinds of magnetic NPs; for instance, cobalt ferrite (CoFe_2_O_4_) NPs, which are of high interest for practical applications due to high coercivity [34] and chemical stability [35]. Current methods of the synthesis of anisotropic cobalt ferrite NPs are rather complex and involve additional compounds [36,37,38]. For instance, 25 nm × 120 nm CoFe_2_O_4_ nanorods were synthesized by a hydrothermal reaction at 130 °C in the presence of cetyltrimethylammonium bromide [38]. Further studies reported the hydrothermal synthesis of cobalt ferrite nanorods doped with Gd^3+^ ions [39] or Pr^3+^ ions [40] in the absence of surfactants. Micrometer-sized cobalt ferrite rods were obtained by thermal decomposition at 400–700 °C of a CoFe_2_(C_2_O_4_)_3_ precursor prepared by a solvothermal reaction [41]. There is only one work in which small (several nm) isotropic cobalt ferrite NPs were assembled into rod-like microaggregates during the thermal decomposition of iron(III) and cobalt(II) acetylacetonates in oleic acid, oleylamine, and a benzyl ether at 200 °C under the gradient magnetic field [42]. However, those microaggregates did not represent single crystals but were composed of individual spherical NPs.

In this article, for the first time, we apply the synthesis in a magnetic field to prepare single-crystal cobalt ferrite nanorods. We evidence that a static magnetic field of 0.4 T is sufficient to obtain single-crystal NPs with a length of 25 nm and a diameter of 3.4 nm, which exhibit stronger superparamagnetic properties than the corresponding isotropic NPs. One can expect that such magnetic-field-assisted 1D growth may be further applied to other magnetic metal oxides as well.

## 2. Materials and Methods

### 2.1. Materials

Iron(III) chloride hexahydrate (FeCl_3_·6H_2_O, purity > 97%) and cobalt(II) nitrate hexahydrate (Co(NO_3_)_2_·6H_2_O, purity > 98%) were purchased from Sigma-Aldrich (Steinheim, Germany). Sodium hydroxide (purity > 98%, residual water content < 15%) was obtained from Acros (Geel, Belgium). All chemicals were used without further purification. The solutions were prepared in distilled deionized water purified by the Millipore Milli-Q system (Burlington, MA, USA).

### 2.2. Synthesis of NPs

Synthesis of NPs was performed by a precipitation reaction of Fe^3+^ and Co^2+^ ions in an alkaline solution. The solution of ions with a Fe^3+^:Co^2+^ molar ratio of 2:1 was prepared by dissolving 2 M FeCl_3_ and 1 M Co(NO_3_)_2_ in water at magnetic stirring. A total of 2 mL of this solution was put into a reaction vessel kept at 70 °C [43] in the presence or absence of a static magnetic field of 0.4 T created by a permanent NdFeB magnet. Then, 2.5 mL of 6.5 M NaOH was added to the reaction medium, and the reaction was allowed to proceed for 4 h. At the end of the reaction, pH was ca. 6.3, which was measured with a MettlerToledo SevenMulti pH meter(Columbus, OH, USA). To increase the magnetic properties, the solution of NPs was aged at 80 °C. The aging time varied from 1 to 250 h. The final product was separated from the liquid by magnetic decantation and washed with distilled water. The purification was repeated 3 times.

### 2.3. Transmission Electron Microscopy

For TEM and high-resolution (HR) TEM measurements, the samples were diluted 10 times with distilled water and after that sonicated for 40 min in pulse mode (5 s of pulses followed by 5 s of rest) with a Sonics VCX 500 ultrasonicator (Newtown, CT, USA) to break the aggregates of NPs. Then, 10 μL of the NP solution was placed onto a 140 mesh Formvar-coated copper grid and air-dried for 3 min at 25 °C. TEM images were obtained using a JEM 2100 F/Cs (Jeol, Tokyo, Japan) operated at 200 kV and equipped with a UHR pole tip as well as a spherical aberration corrector (CEOS, Heidelberg, Germany) and an EEL spectrometer (Gatan, Munich, Germany). The details of the measurements are described elsewhere [44]. The electron micrographs were processed by ImageJ software version 1.54i in order to obtain distances between the crystal planes and to plot the histograms with the NP size distribution [45].

### 2.4. X-ray Diffraction

The crystal structure of the samples was determined by X-ray diffraction using a Bruker AXS D8 DISCOVER setup with a Cu Kα (wavelength λ = 0.15418 nm) radiation at θ-2θ geometry at room temperature. For measurements, samples were prepared by drying the NP solution on a monocrystalline Si (111) wafer.

### 2.5. Raman Spectroscopy

The crystal structure and phase composition of the NPs were investigated using a micro-Raman spectrometer LabRam HR800 (Horiba Jobin Yvon, Villeneuve d’Ascq, France) with an ×100 magnification objective (numerical aperture of 0.9). Details of the experimental procedures are described elsewhere [46]. Measurements were conducted at room temperature in the air environment. A He-Ne laser with a 632.8 nm wavelength was used to excite Raman scattering. The irradiation power density on the sample was continuously decreased until no further changes were observed in the spectra obtained. We found that a power of approximately 0.5 mW and a laser spot diameter of about 10 μm were sufficient to avoid structural changes or phase degradation in the films. The spectra were recorded in the range of 100–800 cm^−1^, and in our measurement conditions, the total acquisition time to obtain a spectrum with a good signal-to-noise ratio was several hours.

### 2.6. X-ray Photoelectron Spectroscopy

The chemical state of the elements in NPs was analyzed by X-ray photoelectron spectroscopy (XPS) on an Axis Ultra DLD spectrometer (Kratos Analytical, Manchester, UK) with the monochromatic Al Kα X-ray source (1486.7 eV, 150 W) under ultra-high vacuum conditions (10^−9^ mbar). Pass energies of 160 and 40 eV were used, respectively, for survey spectra and high-resolution scans. The powder samples were fixed on a holder using non-conductive double-sided adhesive tape. The Kratos charge neutralizer system was used, and the spectra were charge referenced to give the lattice oxygen component in the O1s spectra a binding energy of 530.1 eV, which is typical for iron oxides [47]. This led to the binding energy of the C1s peak, which is characteristic of adventitious carbon (about 285.0 eV), which confirmed the reliability of the charge reference procedure.

### 2.7. Magnetometry

The dependencies of magnetization *M* vs. the applied field strength *H* of the NPs were measured with a vibrating sample magnetometer, LakeShore 7407 (Westerville, OH, USA) (VSM), at 300 K. The strength of the applied magnetic field varied from −16 to 16 kOe. The samples were dried at room temperature and demagnetized before the measurements. The mean values of saturation magnetization *M_s_* and coercive force *H_c_* were deduced from 3 different measurements.

## 3. Results and Discussion

**Figure 1:**
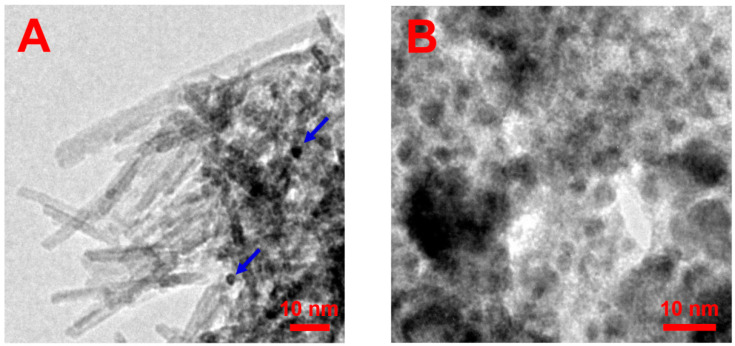
TEM micrographs of NPs synthesized by the co-precipitation of Fe^3+^ and Co^2+^ ions at 70 °C under a magnetic field of 0.4 T (**A**) and in the absence of a magnetic field (**B**). Arrows point out several spheres co-existing with nanorods.

**Figure 2:**
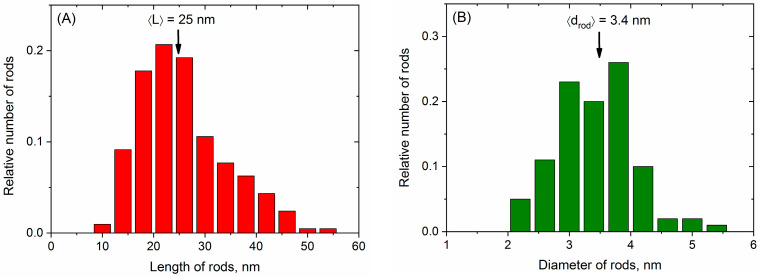

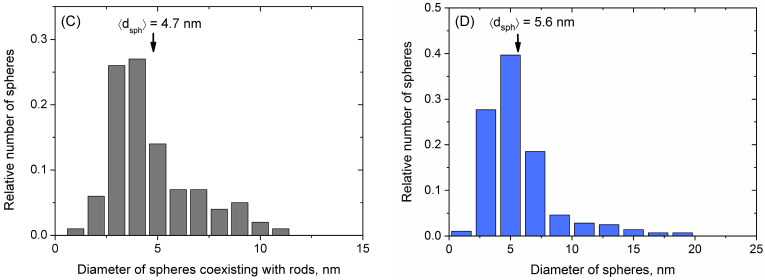
Size distribution histograms of NPs obtained from TEM micrographs: length (**A**) and diameter (**B**) of nanorods synthesized under magnetic field; diameter of spheres co-existing with nanorods (**C**); diameter of NPs synthesized in the absence of magnetic field (**D**). The numbers indicated in the diagrams are relative to the total number of particles analyzed (which was between 100 and 200).

In this study, we have synthesized NPs by the co-precipitation of Fe^3+^ and Co^2+^ ions in an alkaline medium. Under a magnetic field of 0.4 T, the elongated rod-like NPs were obtained (Figure 1A). According to TEM data, their mean length <L> and diameter <d_rod_> are equal to 25 and 3.4 nm, respectively (Figure 2A,B), which corresponds to a rather high aspect ratio of ca. 7. The nanorods co-exist with some spheres indicated by arrows in Figure 1A. The diameter of spheres is ca. 4.7 nm, which is close to that of the nanorods (Figure 2B,C). Previously, the co-existence of spherical NPs with nanorods, grown in the magnetic field, was reported for systems containing magnetite Fe_3_O_4_ [30,32].

The presence of a magnetic field is a principal factor in obtaining the nanorods. Indeed, under the same conditions but in the absence of a magnetic field, there are only isotropic spherical NPs (Figure 1B) with mean diameters of <d_sph_> = 5.6 nm (Figure 2D). This is consistent with the literature data, where the size of the prepared spherical NPs in the sub-10 nm range by the co-precipitation of Fe^3+^ and Co^2+^ was reported to be dependent on the reaction conditions, e.g., temperature [43,48].

**Figure 3:**
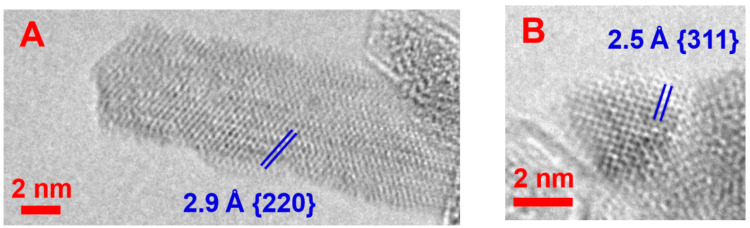

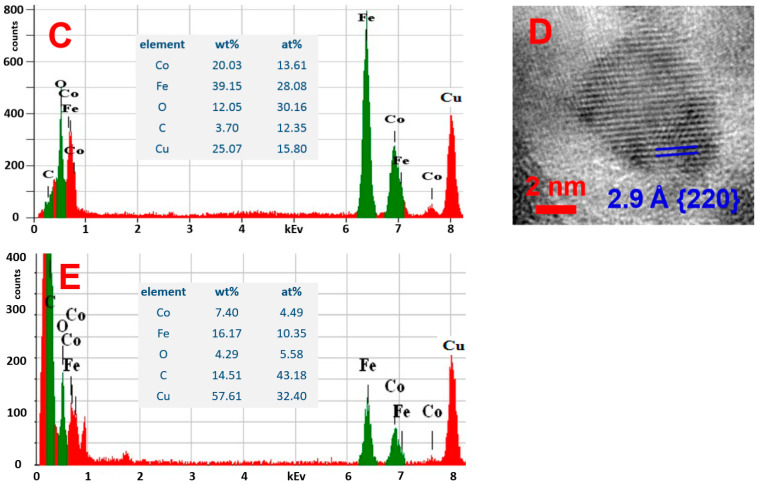
(**A**,**B**) HR TEM micrographs of NPs synthesized under a magnetic field of 0.4 T: (**A**)—nanorod; (**B**)—sphere; (**C**) energy-dispersive X-ray (EDX) spectrum for the sample synthesized under a magnetic field; (**D**) HR TEM micrograph of an NP synthesized in the absence of a magnetic field; (**E**) energy-dispersive X-ray (EDX) spectrum for the sample synthesized in the absence of a magnetic field. In the HR TEM pictures, crystallographic planes and plane spacing distances of cobalt ferrite are identified.

The crystal structure of the synthesized NPs was investigated by TEM. It was shown that the nanorods obtained under a magnetic field are single crystalline (Figure 3A), and the {220} crystallographic planes of cobalt ferrite [49] with a characteristic plane spacing of 2.9 Å can be identified at the micrograph. Isotropic NPs co-existing with nanorods are also single crystals, and {311} planes with spacings of 2.5 Å [50] are seen (Figure 3B). The energy dispersive X-ray (EDX) spectrum shows that the ratio of Fe to Co atoms in the synthesized NPs is close to 2 (Figure 3C), which corresponds to that in the CoFe_2_O_4_ phase (C and Cu peaks at the EDX spectrum arise due to the substrate used in TEM measurements). Thus, HR TEM results show that the co-precipitation of Fe^3+^ and Co^2+^ ions in the magnetic field results in obtaining cobalt ferrite single nanocrystals. Note that NPs synthesized in the absence of a magnetic field are also single crystalline (Figure 3D).

**Figure 4:**
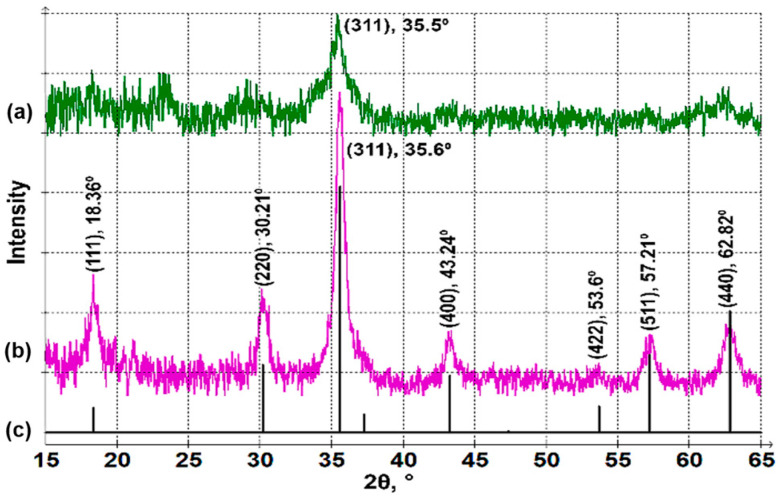
XRD patterns of CoFe_2_O_4_ NPs synthesized in the absence (**a**) and presence (**b**) of a magnetic field and calculated for Co_3−x_Fe_x_O_4_ powder (**c**).

**Figure 5:**
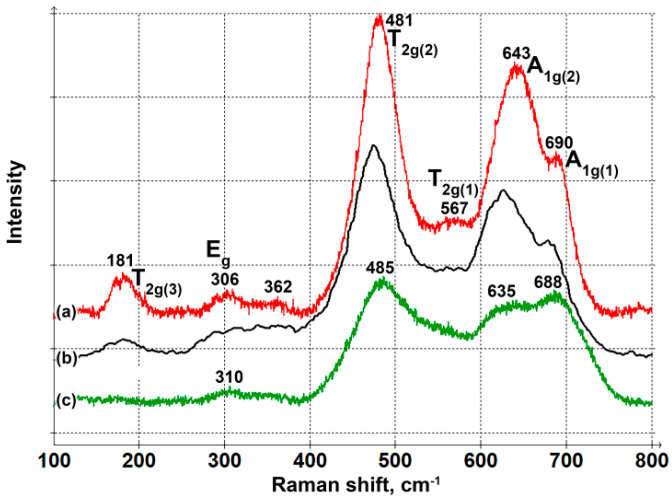
Raman spectra for cobalt ferrite NPs synthesized in the presence (**a**) and absence (**c**) of a magnetic field; Raman spectrum for CoFe_2_O_4_ nanoparticles [54] (**b**); Reproduced with permission from [54], ACS Publications, 2009.

In order to identify the phase composition of the synthesized NPs, XRD (Figure 4) and Raman spectroscopy (Figure 5) were employed. The XRD pattern of the NPs synthesized in the magnetic field (Figure 4b) is consistent with a powder diffraction pattern of a Co_3−x_Fe_x_O_4_ spinel structure (Figure 4c), which was calculated using Profex 5.2.7 software [51] based on a crystal structure from Ref. [52] with a lattice constant of a = 8.36 Å. This lattice constant confirms the composition with x ≈ 2 (for pure CoFe_2_O_4_, a = 8.381 Å, for pure Co_2_FeO_4_, a = 8.242 Å) [52], proving the formation of the CoFe_2_O_4_ inverse spinel. Isotropic particles prepared without a magnetic field produce a much weaker pattern with broader lines (Figure 4a), which is due to the small size of NPs. However, the most intensive (311) spinel reflection at 2θ ≈ 35.5° is also detected. A slight shift of the diffraction peak to lower angles is observed for the spheres (synthesized without magnetic field) compared to the rods (obtained in magnetic field). This may be due to a change in composition [52] or to structural strains in the samples [53]. We believe that the shifts of XRD peaks are due to the minor changes in the elemental composition of the crystals, which is confirmed by XPS data (presented below) showing that cobalt ferrite spheres have a slightly higher content of iron than nanorods (note that the EDX data in Figure 3 show the same trend—the atomic ratio Fe:Co is 2.06 for rods and 2.31 for spheres). A higher amount of iron increases the lattice constant, so XRD peaks shift to lower 2θ values.

The Raman spectrum of rod-like NPs (Figure 5a) is in perfect agreement with that reported for CoFe_2_O_4_ nanoparticles in the literature [54] (Figure 5b). The peaks at 643 and 690 cm^−1^ correspond to the highest frequency A1g mode split into two due to cation inversion [55,56]. These modes involve the symmetric stretching of an oxygen atom with respect to a metal ion in a tetrahedral void. The modes below 600 cm^−1^ involve symmetrical or asymmetrical bending of metal–oxygen bonding in octahedral sites. They have T2g (567, 481, and 181 cm^−1^) or Eg (306 cm^−1^) symmetries. The spectrum of NPs synthesized in the absence of a magnetic field has broader lines, which is due to the small NP size, but the main bands are visible and coincide with the CoFe_2_O_4_ structure. Therefore, XRD and Raman spectroscopy confirm that the NPs synthesized both in the presence and absence of a magnetic field have a spinel CoFe_2_O_4_ structure.

**Figure 6:**
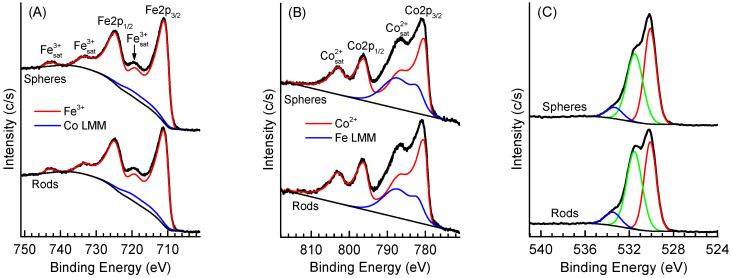
High-resolution Fe2p (**A**), Co2p (**B**), and O1s (**C**) XPS spectra of NPs.

The high-resolution XPS spectra of NPs are presented in Figure 6. Overlapping with Co LMM and Fe LMM Auger lines complicates the analysis of the Fe 2p and, especially Co2p spectra. Both the Fe2p (Figure 6A) and Co2p (Figure 6B) spectra are virtually the same for the rods and spheres. The Fe2p spectra (Figure 6A) show a doublet of the Fe2p_3/2_ and Fe2p_1/2_ lines at binding energies of 711.1 and 724.6 eV with a series of shake-up satellites separated for about 8 and 18 eV from the main lines. Both the position and the satellite structure of the Fe2p spectra are typical for Fe^3+^ species in iron oxides [47,57]. The synthetic component proposed for Fe^3+^ species in [58] fits the Fe2p spectra of NPs. The Co 2p XPS spectra of the samples demonstrate well-pronounced shake-up satellites shifted for about 6.5 eV to higher binding energies from the main Co2p_3/2_ and Co2p_1/2_ lines located at 780.5 and 796.3 eV. These spectra are typical for Co^2+^ species in cobalt oxides [59]. The O1s spectra (Figure 6C), along with the lattice oxygen peak at 530.1 eV, contain two additional peaks. The peak at 531.5 eV can be attributed to surface hydroxide and carbonate species, while the peak at 533.5 eV can be assigned to single O−C bonds in adventitious carbon on the surface. Such oxygen spectra are typical for the ex situ prepared metal oxide samples. Summarizing, the XPS data confirm that both samples mainly contain Fe^3+^ and Co^2+^ species coordinated with lattice oxygen.

**Figure 7:**
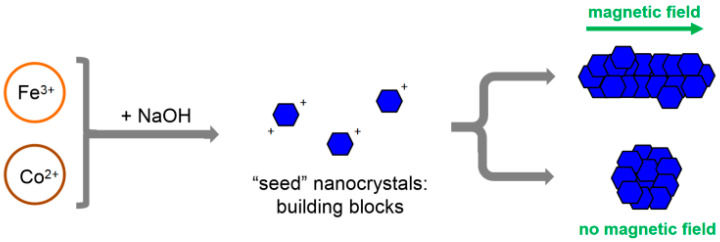
Schematic representation of the growth mechanism of cobalt ferrite nanorods in the presence and absence of a magnetic field.

These results suggest a mechanism for the anisotropic growth of rod-like cobalt ferrite NPs in the magnetic field (Figure 7), which is similar to the mechanism proposed earlier for the magnetic-field-assisted synthesis of rod-like magnetite [32]. At the first stage of the co-precipitation reaction, small isotropic “seed” NPs are formed. At this stage, most of the OH^−^ ions of alkali are consumed for the formation of cobalt ferrite according to the following reaction:

Co^2+^ + 2Fe^3+^ + 8 OH^−^ → CoFe_2_O_4_ + 4H_2_O (1)

This induces a drop in pH from an initial value of ~14 to ~6.3, which was measured for the syntheses carried out in the present work. CoFe_2_O_4_ has an isoelectric point of ca. 7 [60]; therefore, at pH 6.3, the “seed” NPs are only slightly positively charged. Thus, electrostatic repulsion between the “seeds” is not strong enough to prevent self-assembly into columnar structures due to magnetization obtained in the external magnetic field [61]. At the latter reaction stages, the “seeds”, which are assembled together, fuse into a single-crystalline rod. The growth of rods by aggregation and the re-crystallization of primary particles in the magnetic field was previously described for micrometer-sized rod-like magnetite particles synthesized by co-precipitation [28]. This resembles the general features of the nanoparticles’ growth [62]. In the absence of a magnetic field, the “seeds” do not fuse into rods but into some larger isotropic NPs (Figure 7).

**Figure 8:**
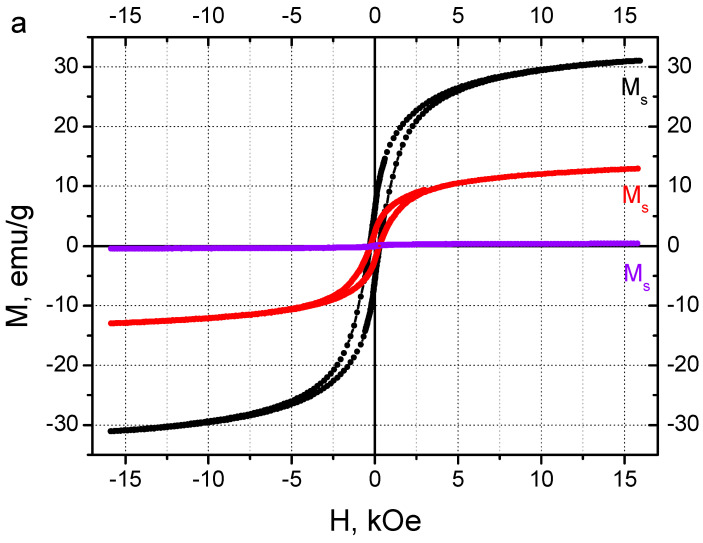

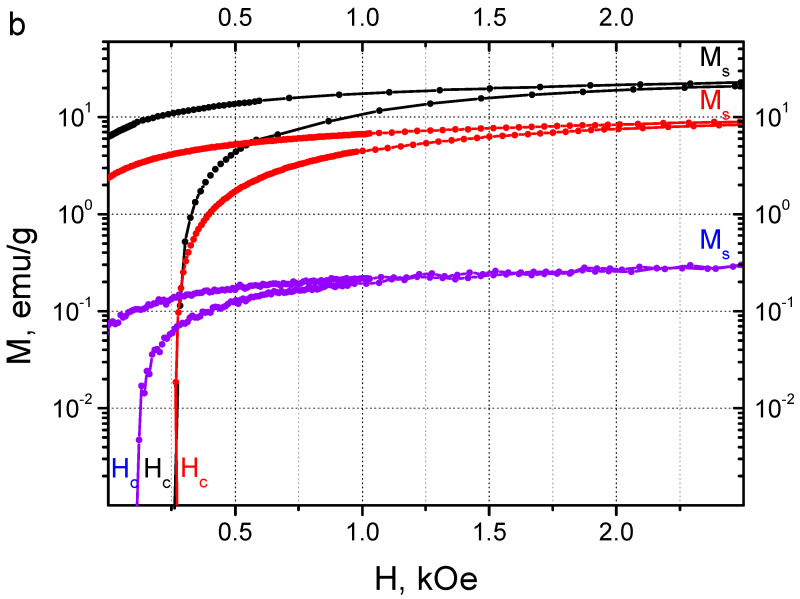
Magnetic hysteresis loops of isotropic cobalt ferrite NPs (violet) and anisotropic cobalt ferrite nanorods before (red) and after (black) aging for 1 h at 80 °C (**a**); the enlargement of the loops at low strength of the applied magnetic field (**b**).

The magnetic properties of synthesized cobalt ferrite NPs were studied with magnetometry. In Figure 8a, the hysteresis curves, obtained from the VSM measurements at room temperature (300 K), are depicted. The values of saturation magnetization *M_s_* and coercivity *H_c_* determined from the curves (Figure 8b) are presented in Table 1. In Figure 8a, one can see that the size of the hysteresis loop is much larger for nanorods. As a consequence, the sample containing nanorods demonstrates significantly higher values of *M_s_* and *H_c_* (Figure 8b), which indicates that nanorods possess stronger magnetic properties than isotropic NPs. The rise of saturation magnetization and coercivity with the size of monocrystalline CoFe_2_O_4_ NPs was previously described in the literature [63]. It was explained by increasing the size of magnetic domains where the atomic spins are aligned along the direction of the applied magnetic field. As a result, the maximum magnetization of the particles and the strength of the reverse external field required to demagnetize them increased [63].

Obtained nanoparticles are single crystals (Figure 3A), and they have slightly lower but comparable magnetic properties compared to larger polycrystalline particles [35] and bulk CoFe_2_O_4_ [64]. An increase in the nanocrystal size induces a rise of saturation magnetization, reaching 80 emu/g for 50 nm NPs and not increasing further up to the bulk. The coercive force shows a non-linear dependence on the NP size. A maximum of 1.2 kOe is reached for 25 nm NPs, and it is lower for both smaller (as in this work, where it equals 0.6 kOe) and larger NPs. For the bulk, the coercive force is only 0.06 kOe.

**Figure 9:**
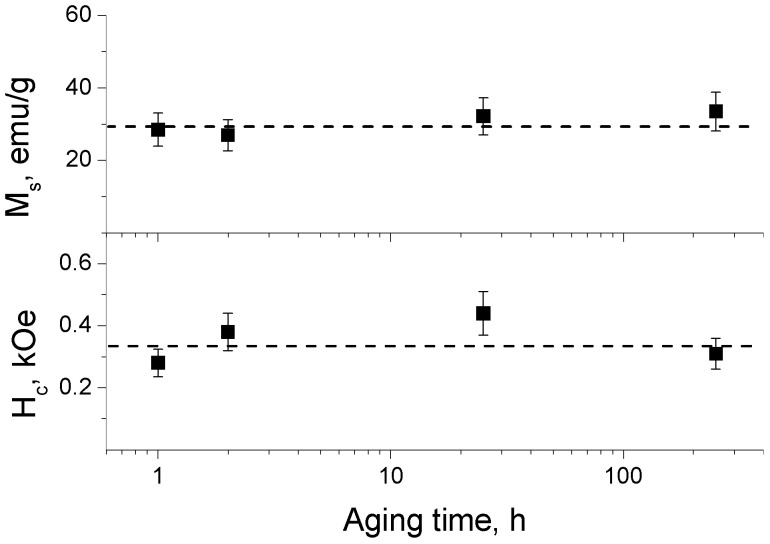
Dependences of saturation magnetization *M_s_* and coercivity *H_c_* of cobalt ferrite nanorods on aging time at 80 °C.

The solution of synthesized nanorods was aged at 80 °C to increase the magnetic properties [65]. The hysteresis loop of the nanorods after aging for 1 h demonstrates a 3 times higher value of saturation magnetization *M_s_*, than nanorods before aging (Figure 8a, Table 1). The increase in *M_s_* upon aging can be attributed to the increased size of the crystallite, as was suggested by F. Huixia and co-authors [65] for aged isotropic CoFe_2_O_4_ NPs. Unlike *M_s_*, the value of coercivity *H_c_* of the nanorods before and after aging remains almost the same (Table 1). Probably, the temperature of aging is not high enough to additionally adjust the aligned atomic spins inside the rod-like particles. Longer aging up to 250 h (Figure 9) does not affect the *M_s_* and *H_c_* values (see Supplementary Information for the corresponding hysteresis loops). Therefore, 1 h of aging is enough to obtain anisotropic CoFe_2_O_4_ NPs with maximum saturation magnetization *M_s_* and coercivity *H_c_*, which are equal to 30 ± 1 emu/g and 330 ± 15 Oe, respectively.

## 4. Conclusions

In this paper, it is shown, for the first time, that a static magnetic field of moderate strength can be used as a template for the anisotropic growth of cobalt ferrite NPs, which is one of the most attractive nanomaterials in many applications. Nanorods with an aspect ratio of ca. 7 are synthesized by the co-precipitation of Fe^3+^ and Co^2+^ ions in alkali. They are single crystals and co-exist with some spherical NPs. EDX, XRD, and Raman spectroscopy confirm the formation of a CoFe_2_O_4_ spinel structure. The magnetic field is demonstrated to be a key factor that provides anisotropic growth at the same synthesis conditions. But, in the absence of a magnetic field, only small isotropic CoFe_2_O_4_ NPs are obtained. It was suggested that anisotropic cobalt ferrite NP growth proceeds in two steps: (1) the formation of “seed” NPs and (2) the self-assembly of “seeds” into columns in the magnetic field and their fusion into single-crystal nanorods. According to VSM data, the maximum values of saturation magnetization Ms and coercivity Hc of the nanorods are obtained after 1 h of aging at 80 °C and equaled 30 emu/g and 0.33 kOe, respectively. The results of this paper demonstrate that a facile and “green” magnetic-field-assisted synthesis of anisotropic NPs with pronounced magnetic properties can be applied not only to magnetite but also other metal oxides. This opens a route for simplifying the production of anisotropic magnetic nanoparticles, which are currently synthesized mostly by rather complex methods. The surface of the obtained nanorods is free of additional stabilizers and can be further easily modified. Cobalt ferrite nanorods may be used in various practical applications, including biomedicine, energy storage, green energy production, and the preparation of magnetic nanocomposites and gels with controlled anisotropy.

## Figures and Tables

**Table 1:** Values of saturation magnetization *M_s_* and coercivity *H_c_* anisotropic cobalt ferrite nanorods before and after 1 h of aging at 80 °C in comparison with isotropic cobalt ferrite NPs.

	*M_s_*, emu/g	*H_c_*, Oe
Isotropic CoFe_2_O_4_ NPs	0.3	111
CoFe_2_O_4_ nanorods	8.3	248
CoFe_2_O_4_ nanorods after 1 h of aging	28.5	265

## Data Availability

The data presented in this study are openly available.

## Supplementary Materials

The following supporting information can be downloaded at https://www.mdpi.com/article/10.3390/nano14060541/s1, Figure S1: hysteresis loops of cobalt ferrite nanorods after aging for different periods of time at 80 °C.
