# Supplementary material for: Cobalt Ferrite Nanorods Synthesized with a Facile “Green” Method in a Magnetic Field
Source: Nanomaterials (Basel). 2024 Mar 20;14(6):541. doi: 10.3390/nano14060541 (PMC10976011; doi:10.3390/nano14060541)
Supplement: Supplementary file 1 [file nanomaterials-14-00541-s001.zip › nanomaterials-2907303-supplementary.pdf]

Supplementary Information

for

Cobalt Ferrite Nanorods Synthesized with a Facile "Green" Method in Magnetic Field

Alexander L. Kwiatkowski, Petr V. Shvets, Ivan S. Timchenko, Darya E. Kessel, Elizaveta D. Shipkova, Konstantin I. Maslakov, Ivan A. Kuznetsov, Dmitry A. Muravlev, Olga E. Philippova and Andrey V. Shibaev

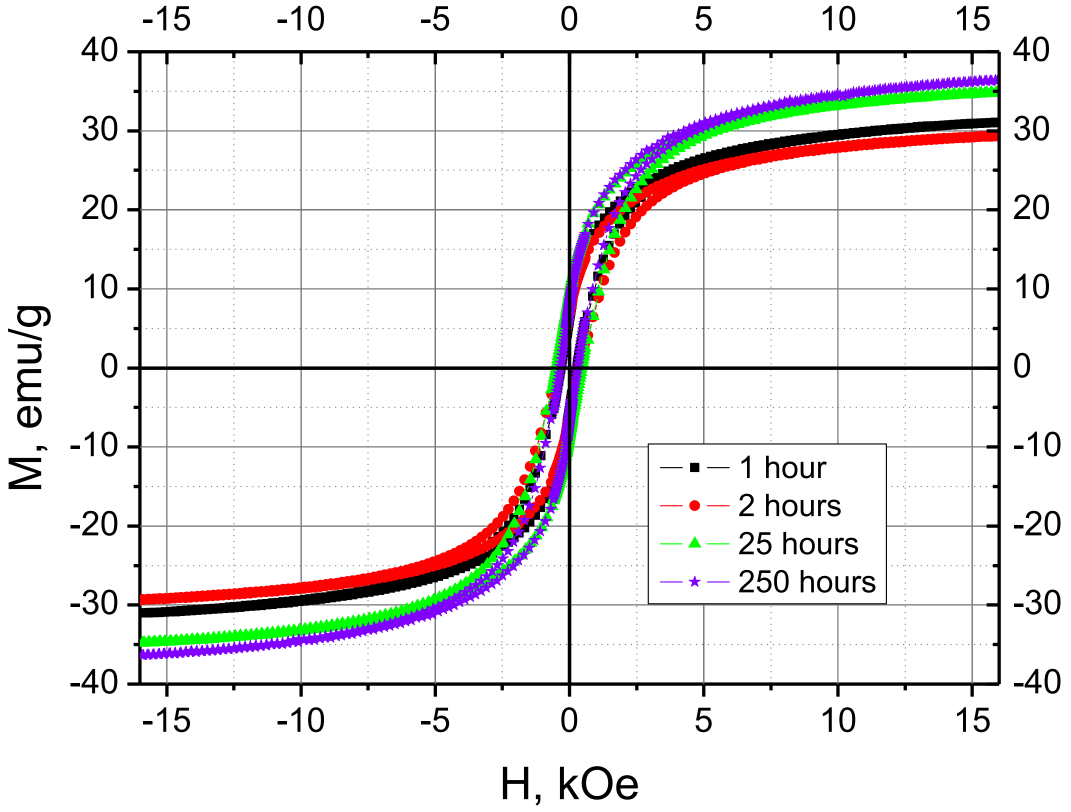

Figure S1. Hysteresis loops of cobalt ferrite nanorods after aging for different periods of time at 80°C.
